# Correcting promoter and beta-lactamase ORF orientation in a widely-used retroviral plasmid to restore bacterial growth

**DOI:** 10.1038/s41598-025-93222-y

**Published:** 2025-03-11

**Authors:** Jürgen Wittmann

**Affiliations:** Division of Molecular Immunology, Department of Internal Medicine III, Nikolaus-Fiebiger-Center of Molecular Medicine (NFZ), Friedrich-Alexander-Universität Erlangen-Nürnberg (FAU), Erlangen, Germany

**Keywords:** pBMN-I-GFP, Antibiotic resistance, Ampicillin promoter, *E. coli*, Plasmid transformation, Retroviral functionality, Cell biology, Immunology, Molecular biology

## Abstract

The pBMN-I-GFP plasmid is a widely used retroviral vector for producing retroviral particles, utilized by thousands of laboratories worldwide. However, we observed that *E. coli* transformed with pBMN-I-GFP failed to grow on selective LB agar plates containing ampicillin or carbenicillin, in contrast to similar retroviral vectors. Multiple attempts to optimize growth conditions were unsuccessful. Sequencing, contrary to the available reference sequence, revealed an inversion of the beta-lactamase (*bla*) gene and part of its promoter, likely disrupting *bla* expression and, consequently, antibiotic resistance. To address this, we corrected the orientation of the ampicillin resistance gene and its promoter in a new plasmid, prBMN-I-EGFP. This modification restored robust growth of *E. coli* transformed with this plasmid on selective plates, confirming the essential role of an intact *bla* promoter for antibiotic resistance. Additionally, retroviral functionality tests in murine cell lines showed that prBMN-I-EGFP exhibited transfection and infection efficiencies comparable to the original pBMN-I-GFP. These findings underscore the importance of thorough sequence verification for commonly used plasmids and present an improved version of pBMN-I-GFP.

**Supplementary Information:**

The online version contains supplementary material available at 10.1038/s41598-025-93222-y.

## Introduction

Transfection is a fundamental technique in molecular biology for introducing nucleic acids into cells. However, many cell types, particularly primary and immune cells, are notoriously difficult to transfect efficiently. In these cases, retroviral infection offers a highly effective alternative. Retroviral vectors use reverse transcriptase to convert single-stranded RNA into double-stranded DNA, allowing stable integration into the host genome. This feature supports long-term gene expression, which is critical for extended experimentation and analysis. In addition, retroviral vectors excel at delivering large genetic payloads to dividing cells, making them particularly valuable for gene delivery in mammalian systems.

In the laboratory, retroviral vectors are designed to safely deliver genes of interest without spreading infection. A retroviral plasmid construct, containing the desired gene, is introduced into a packaging cell line. This cell line supplies essential viral proteins, such as gag-pol and envelope, necessary for assembling infectious retroviral particles. These particles are released into the culture medium, collected, and used to infect target cells. Importantly, because the packaging cell line does not transfer the viral genes responsible for replication, the transduced cells cannot propagate the virus, ensuring safety during experimentation.

One of the most widely adopted retroviral packaging systems is the Phoenix cell line, developed by the Garry Nolan lab and distributed to thousands of laboratories globally (Nolan Laboratory Retroviral Systems. Phoenix helper-free retrovirus producer lines, https://web.stanford.edu/group/nolan/_OldWebsite/retroviral_systems/retsys.html, accessed on October 23, 2024). Derived from human embryonic kidney (HEK) 293T cells, Phoenix cells are engineered to stably express the key retroviral packaging components necessary for producing high-titer, replication-incompetent retroviruses. These cells facilitate efficient gene transfer with both ecotropic and amphotropic retroviral particles, making them a versatile tool in gene delivery systems.

In addition to the Phoenix cells, the Nolan lab has distributed several foundational retroviral plasmids, including pBMN-I-GFP, which is also available through repositories like Addgene. This plasmid, derived from the ecotropic Moloney murine leukemia virus, facilitates the expression of cDNA alongside an EGFP marker, allowing for efficient tracking of gene delivery and expression in transduced cells.

While pBMN-I-GFP has not been formally published, its origins likely trace back to the retroviral vector advancements of the 1980s, a period marked by significant progress in understanding proviral DNA elements from murine retroviruses [reviewed in ^1^. The plasmid’s lineage likely involves early constructs such as pZIP-Neo SV(X)1^2^ and direct orientation (DO) vectors^3^, both of which laid the groundwork for more efficient vector systems. These efforts eventually culminated in the creation of the pBabe vector series^4^. A potential direct precursor of pBMN-I-GFP is the LZRS-LacZ(A) vector, a hybrid of pBabe Puro and the retroviral MFG vector, incorporating the EBNA-1 gene for enhanced functionality^5^.

Despite its widespread use, we observed unexpected growth failures in *E. coli* transformed with pBMN-I-GFP under selective conditions. The *bla* antibiotic resistance cassette present in this plasmid encodes the enzyme β-lactamase, which hydrolyzes the β-lactam ring in antibiotics such as ampicillin and carbenicillin. This enzymatic activity neutralizes the antibiotics, thereby conferring resistance to the carrier strain. However, mutations in the *bla* gene can result in reduced or inhibited growth of the host organism due to its inability to neutralize these antibiotics. While other retroviral plasmids such as pBabe Puro allowed for normal bacterial growth, pBMN-I-GFP repeatedly failed to confer antibiotic resistance, complicating routine bacterial propagation. This inconsistency prompted us to investigate the underlying cause of this issue and determine whether plasmid configuration errors were responsible for the observed phenotype.

In this study, we aimed to identify the cause of the observed growth defect and explore ways to restore antibiotic resistance. Through sequencing analysis and restriction digests, we discovered an inversion within the *bla* open reading frame (ORF) and its associated promoter region, likely disrupting the expression of the *bla* gene. By correcting the orientation of this inverted fragment in a newly designed plasmid, prBMN-I-EGFP, we successfully restored bacterial growth on selective media. Additionally, we verified that this correction did not compromise the plasmid’s retroviral functionality, ensuring its continued utility as a tool for gene delivery in mammalian cells. These findings underscore the importance of validating plasmid sequences and highlight broader considerations in plasmid design for molecular biology research.

## Results

### Failure of ***E. coli*** growth on selective plates after transformation with pBMN-I-GFP plasmid

**Fig. 1:**
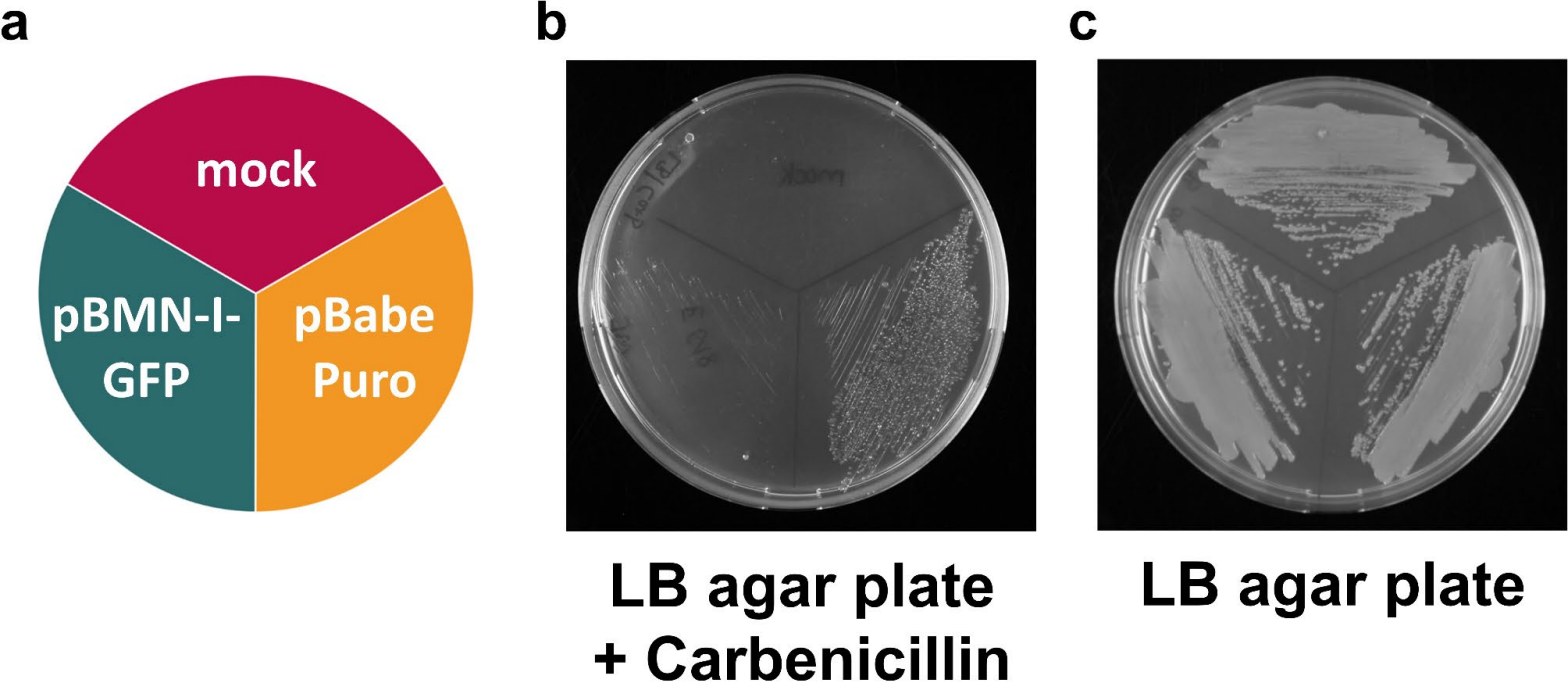
Growth of *E.* *coli* after transformation with retroviral plasmids pBMN-I-GFP and pBabePuro on LB/carbenicillin and LB agar plates. Heat-shock competent *E. coli* Stbl3 cells were transformed with either no plasmid DNA (mock) or with plasmids pBMN-I-GFP or pBabe Puro and streaked as shown in (**a**) onto (**b**) an LB agar plate containing 50 µg/mL carbenicillin or (**c**) an LB agar plate without antibiotics. Plates were incubated at 37 °C for 20 h, and images were captured. All plasmid transformations were performed in the same *E. coli* strain (Stbl3). Results are representative of three independent experiments.

We encountered significant growth difficulties with *E. coli* transformed with the retroviral pBMN-I-GFP plasmid (Supplementary Fig. 1) and its derivatives. Heat-shock transformation of *E. coli* Stbl3 cells with pBMN-I-GFP (schematically depicted in Fig. 1a) resulted in no growth on LB agar plates supplemented with 50 µg/mL carbenicillin after overnight incubation at 37 °C (Fig. 1b). In contrast, similar retroviral vectors such as pBabe Puro^4^ supported normal bacterial growth, while mock-transformed *E. coli* Stbl3 cells, as expected, did not grow on selective plates (Fig. 1b). The post-transformation viability of *E. coli* Stbl3 cells was confirmed by their vigorous growth on LB plates lacking antibiotics (Fig. 1c). Growth was similarly inhibited on LB agar plates containing 100 µg/mL ampicillin (Supplementary Fig. 2), with identical results obtained when using glycerol stocks of transformed bacteria streaked from − 80 °C storage.

Various efforts to optimize growth conditions, such as extended incubation at 37 °C, incubation at room temperature, glycerol supplementation, reduced antibiotic concentrations, or richer growth media (SOB or SOC), failed to significantly improve colony formation or plasmid yield. Similar results were obtained when testing different *E. coli* strains or using electroporation instead of heat-shock transformation. Even when few colonies appeared after 24 h, they exhibited minimal further growth over the next 24 h. Liquid culture inoculation from these colonies in LB medium supplemented with carbenicillin or ampicillin also failed to yield robust turbidity after 24 h, and plasmid recovery after 48 h was either absent or extremely low. As pBMN-I-GFP is meanwhile also available from Addgene (https://www.addgene.org/1736/, see the “Citations” section for manuscripts using this plasmid, accessed on October 22, 2024), we noted in the depositor’s comments the remark, “This plasmid is extremely slow growing and may need 2 days to grow”, which confirmed our own observations (Supplementary Fig. 3).

### Restriction digest and sequence analysis of pBMN-I-GFP

**Fig. 2:**
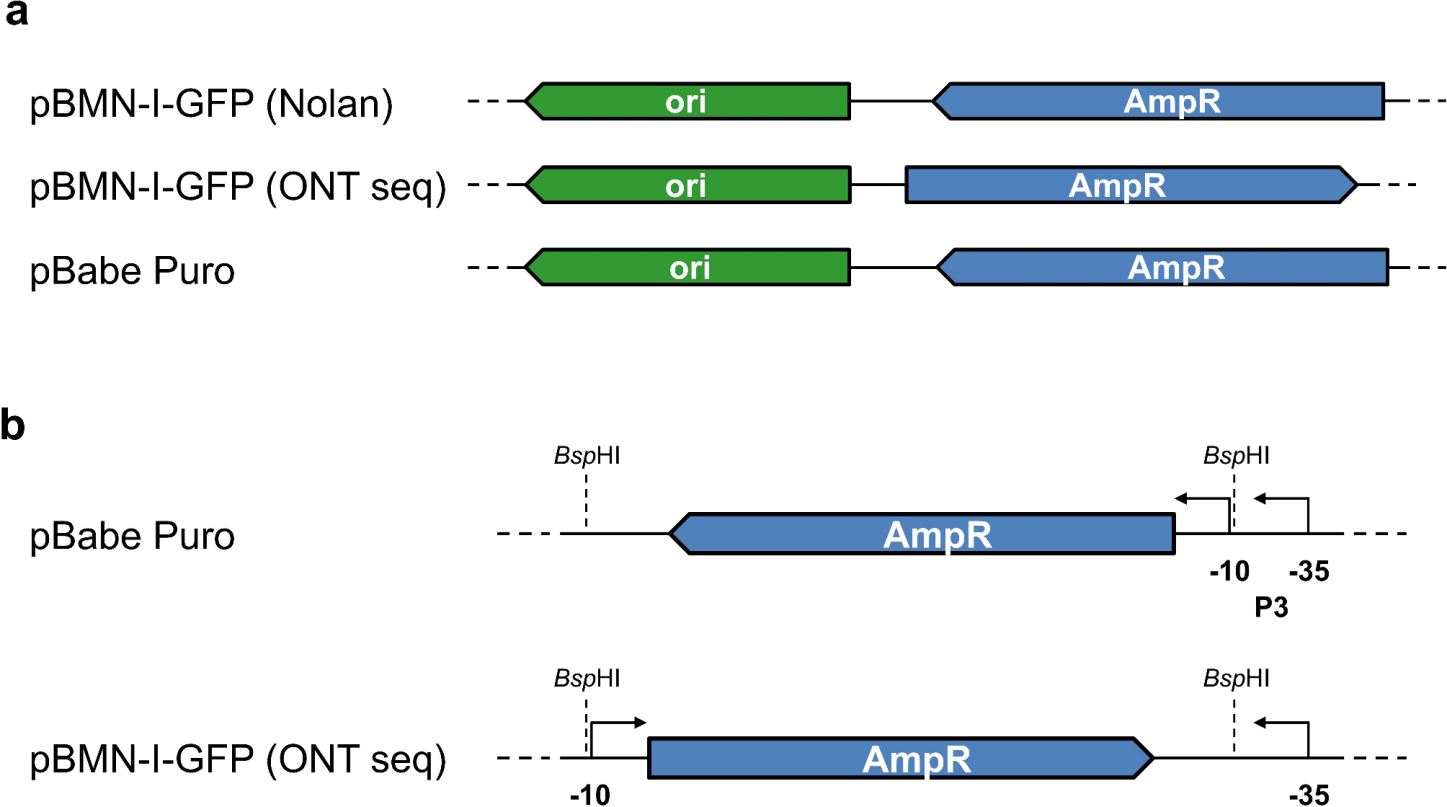
Schematic presentation of the orientation of the Ampicillin ORF in different retroviral plasmids. (**a**) Schematic illustration showing the orientation and positioning of the ampicillin resistance gene (AmpR) relative to other vector components, such as the origin of replication (ori), in the retroviral vectors pBMN-I-GFP according to the Nolan group website sequence [“pBMN-I-GFP (Nolan)”, first line], pBMN-I-GFP after Oxford Nanopore Technology sequencing [“pBMN-I-GFP (ONT seq)”, second line], and pBabe Puro (third line). Figures are drawn to scale. (**b**) A zoomed-in view of the region surrounding the ampicillin resistance gene in “pBabe Puro” (first line) and “pBMN-I-GFP (ONT seq)” (second line). *Bsp*HI: restriction site for *Bsp*HI, P3: promoter 3 of AmpR, -10 and − 35: -10 and − 35 regions of the AmpR promoter 3. Figure is not drawn to scale.

Several restriction digests of pBMN-I-GFP yielded expected results; however, a discrepancy arose when the plasmid was digested with the restriction enzyme *Fsp*I. The provided restriction map for this plasmid, received by us from the Nolan lab in 2002, indicated the presence of two fragments, but only one fragment was observed. Since the *Fsp*I restriction site is neither sensitive for *dam* or *dcm* methylation in *E. coli* and these predicted restriction sites are located within and near the ampicillin resistance gene (AmpR), we decided to perform Oxford Nanopore Technology (ONT) sequencing to determine the precise full plasmid sequence. This analysis revealed that the plasmid is, in fact, 6353 bp in length, contrary to the reported 6332 bp, and only contains one *Fsp*I site. Sequence analysis and alignment using MultAlin^6^ (Supplementary Fig. 4) identified minor nucleotide changes in the LTR regions and upstream of the IRES region, but most notably, the orientation of the AmpR open reading frame (ORF) and adjacent sequences was inverted relative to expectations (Fig. 2a, Supplementary Fig. 5). Given that the pBMN vectors are derived from the pBabe vectors, we also performed ONT sequencing on pBabe Puro, which confirmed the plasmid map as predicted (Fig. 2a, Supplementary Fig. 6).

Since pBMN-I-GFP is unpublished and its genealogy only partially traceable, we performed a detailed analysis of the region surrounding the AmpR ORF. Most AmpR genes in laboratory plasmids are derived from pBR322^7^. Early studies on the transcription of *bla*, the enzyme ultimately responsible for conferring ampicillin resistance, revealed two promoters in pBR322, P1 and P3, which drive *bla* transcription, and a third promoter, P2, which transcribes toward the *tet* gene^8^. In the construction of common cloning vectors such as pUC18/19^9^ or pBluescript SK(-)^10^, the *bla* promoter region underwent significant modifications, with most vectors retaining only the P3 promoter, the native promoter of the *bla* ORF.

Upon examining the *bla* ORF and surrounding sequences in both pBMN-I-GFP and pBabe Puro plasmids, we identified a *Bsp*HI restriction site within the − 10 and − 35 regions of the P3 promoter. A second *Bsp*HI site was also located downstream of the *bla* ORF (Fig. 2b). The use of the *Bsp*HI restriction site during the construction of pBMN-I-GFP may have inadvertently caused an inversion of the *bla* ORF and a significant portion of its promoter. This inversion could potentially disrupt *bla* gene expression, reducing the plasmid’s ability to confer resistance to β-lactam antibiotics, which would impair bacterial growth on LB/carbenicillin agar selection plates (Supplementary Fig. 7).

### Rescue of antibiotic resistance by reversing the orientation of the AmpR gene fragment

To test whether restoring the correct orientation of the AmpR gene fragment would recover antibiotic resistance, we corrected the orientation of the *bla* gene and the P3 promoter in pBMN-I-GFP using the corresponding sequence from pBabe Puro, generating a new plasmid, prBMN-I-EGFP (reflecting the use of the “EGFP” fluorescent protein ORF rather than GFP). ONT sequencing confirmed that the plasmid’s configuration now matched both the initial pBMN-I-GFP map and the pBabe Puro sequence (Supplementary Fig. 8).

**Fig. 3:**
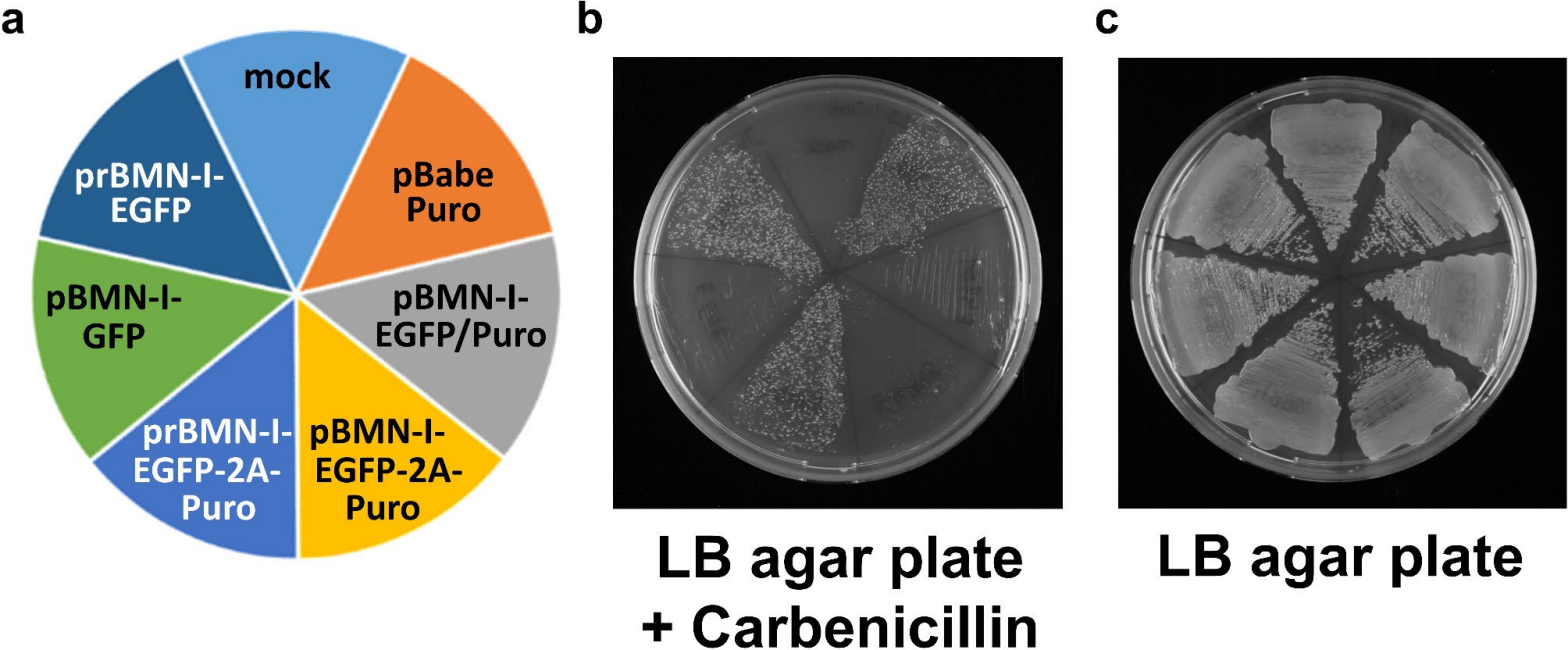
Growth of *E.* *coli* after transformation with various retroviral plasmids on LB/carbenicillin and LB agar plates. Heat-shock competent *E. coli* Stbl3 cells were transformed with either no plasmid DNA (mock) or with retroviral plasmids (pBabe Puro, pBMN-I-EGFP/Puro, pBMN-I-EGFP-2A-Puro, prBMN-I-EGFP-2A-Puro, pBMN-I-GFP and prBMN-I-EGFP) and streaked as shown in (**a**) onto (**b**) an LB agar plate containing 50 µg/mL carbenicillin or (**c**) an LB agar plate without antibiotics. Plates were incubated at 37 °C for 20 h, and images were captured. All plasmid transformations were performed in the same *E. coli* strain (Stbl3). Results are representative of three independent experiments.

When transformed into *E. coli* Stbl3 cells (schematically depicted in Fig. 3a), the prBMN-I-EGFP plasmid enabled significant bacterial colony formation on LB/carbenicillin agar plates, whereas pBMN-I-GFP still failed to confer antibiotic resistance (Fig. 3b). Control cells, transformed with no plasmid DNA (mock), failed to grow, and bacterial viability post-transformation was confirmed by growth on non-selective LB agar plates (Fig. 3c).

To confirm the applicability of these findings to other variants of pBMN-I-GFP, we generated and tested prBMN-I-EGFP-2A-Puro, a version of pBMN-I-EGFP-2A-Puro^11^ with a reversed AmpR fragment (Supplementary Fig. 9). Consistent with the results obtained for prBMN-I-EGFP, prBMN-I-EGFP-2A-Puro restored antibiotic resistance (Fig. 3b), while other derivatives such as pBMN-I-EGFP/Puro and pBMN-I-EGFP-2A-Puro still failed to support bacterial growth on selective media.

To determine whether the observed growth correlates with increased β-lactamase activity, we performed an enzymatic assay. When bacteria produce significant amounts of β-lactamase, a disc impregnated with the yellow chromogenic cephalosporin nitrocefin turns red at the site of bacterial application. This color change signifies hydrolysis of the amide bond in the β-lactam ring, indicating β-lactamase activity.

**Fig. 4:**
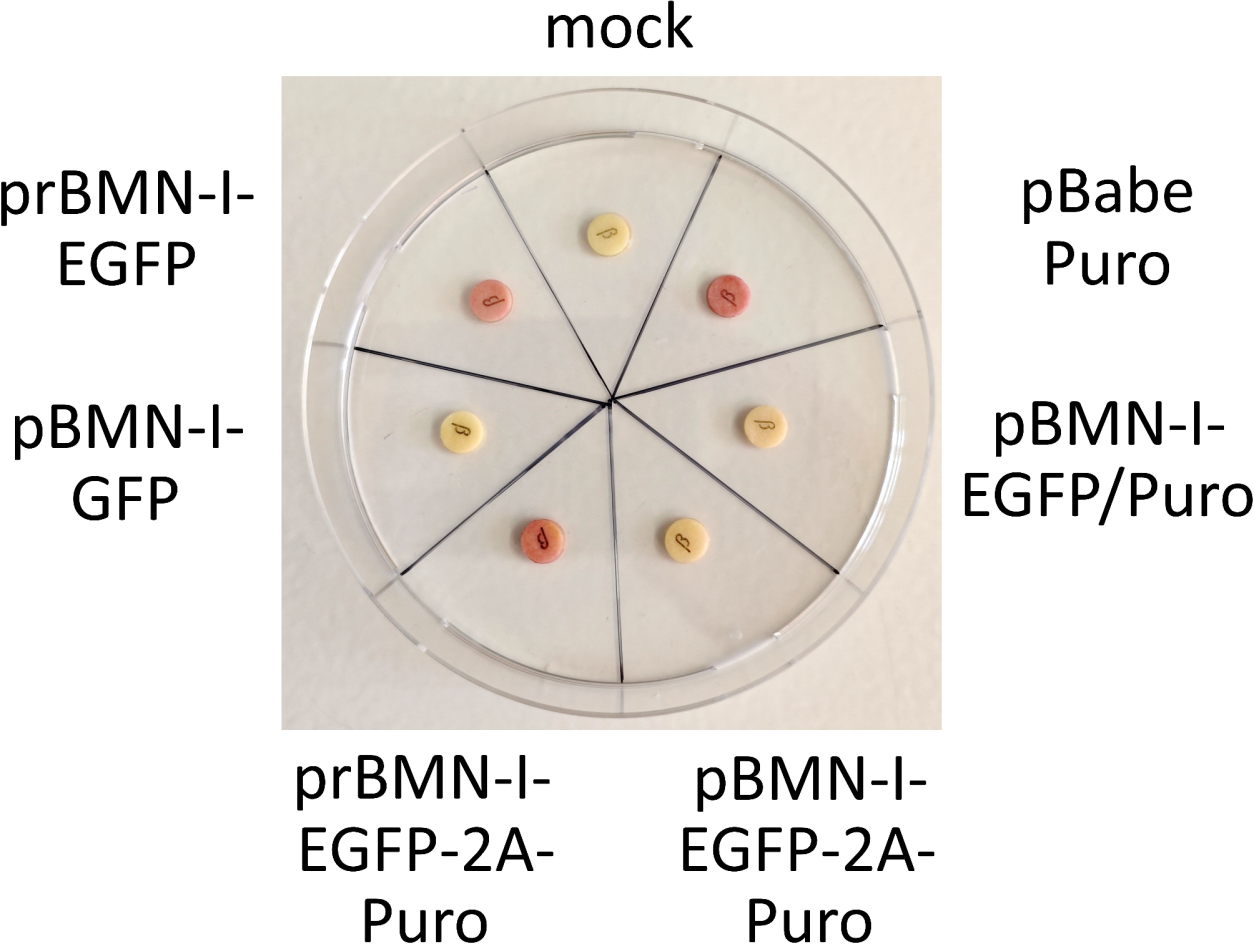
β-Lactamase activity of *E. coli* after transformation with various retroviral plasmids. Heat-shock competent *E. coli* Stbl3 cells were transformed with either no plasmid DNA (mock) or one of the following retroviral plasmids: pBabe Puro, pBMN-I-EGFP/Puro, pBMN-I-EGFP-2A-Puro, prBMN-I-EGFP-2A-Puro, pBMN-I-GFP, or prBMN-I-EGFP. The transformed cells were inoculated into LB medium containing 50 µg/mL carbenicillin and grown at 37 °C to stationary phase. Discs impregnated with the chromogenic cephalosporin nitrocefin were moistened with purified water, and bacterial suspensions were applied to the disc surface to observe for color changes. The results shown are representative of two independent experiments.

*E. coli* Stbl3 cells were heat-shock transformed with the six plasmids described Fig.  3, along with a mock transformation control, and inoculated into liquid LB/carbenicillin medium. After reaching stationary phase, a small volume of bacterial suspension was applied to the nitrocefin disc, and color change was recorded after 5 min. As shown Fig. 4, prBMN-I-EGFP, prBMN-I-EGFP-2A-Puro, and the positive control pBabe Puro caused an immediate red color change, while bacteria transformed with the other plasmids, as well as the mock negative control, remained yellow.

These findings demonstrate that restoring the correct orientation of the *bla* gene and the split P3 promoter effectively rescues the plasmid’s antibiotic resistance by producing detectable levels of β-lactamase, enabling robust growth on LB/carbenicillin agar plates, similar to pBabe Puro. Additionally, these results suggest that the observed effects extend to other derivatives of pBMN-I-GFP.

### Impact of corrected AmpR fragment orientation on retroviral functionality

To assess whether the corrected orientation of the *bla* ORF and P3 promoter affected the retroviral functionality of the plasmids, we infected the murine pro-B cell line 38B9^12^ with retroviral supernatants generated from pBMN-I-GFP and prBMN-I-EGFP. Retroviral particles were produced in Platinum-E packaging cells^13^, and retroviral supernatants were harvested 72 h post-transfection. Flow cytometric analysis of Platinum-E cells revealed comparable transfection efficiencies for both pBMN-I-GFP and prBMN-I-EGFP (Supplementary Fig. 10).

**Fig. 5:**
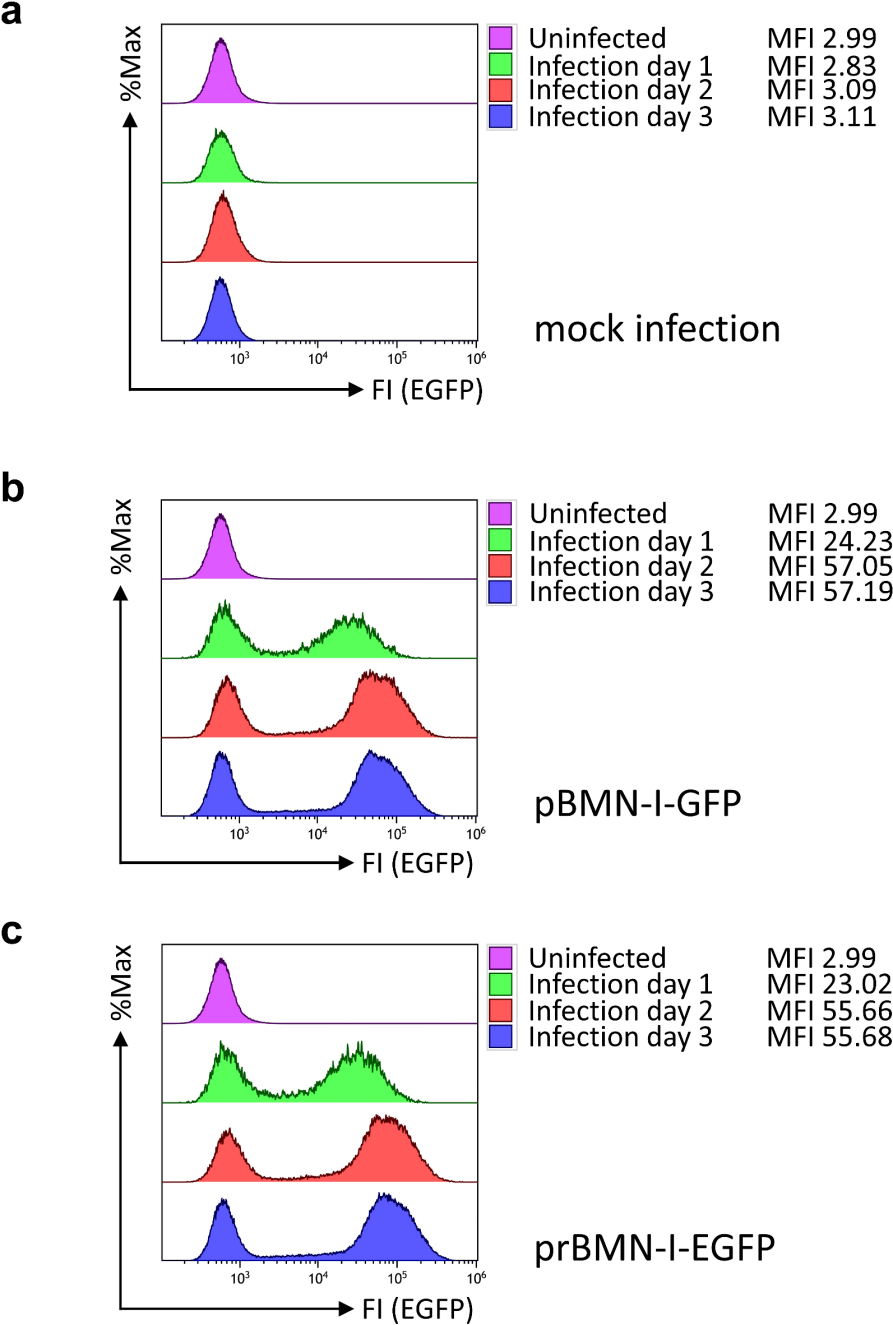
Flow cytometric analyses for EGFP fluorescence in 38B9 cells infected with retroviral supernatants derived from pBMN-I-GFP and prBMN-I-EGFP. 38B9 cells were infected with retroviral supernatants from (**a**) mock-, (**b**) pBMN-I-GFP- or (**c**) prBMN-I-EGFP-transfected Platinum-E cells. EGFP fluorescence intensities, used to evaluate infection efficiency, were measured by flow cytometry on the day of infection, one day after infection, and every other day for 3 days. Data acquisition and gating strategy followed those described in Supplemental Fig. 11. EGFP fluorescence intensities of live, single cells are presented as overlay histograms, with relative cell numbers normalized and presented as %Max. The mean fluorescence intensities (MFIs) of the EGFP-positive populations are indicated next to the labels. The results are representative of two independent experiments, each performed using retroviral supernatant generated from independently isolated plasmids.

Retroviral infection of 38B9 cells via spinoculation with supernatant from mock-transfected Platinum-E cells did not produce any EGFP-positive cells (Fig. 5a). In contrast, supernatant from pBMN-I-GFP and prBMN-I-EGFP transfected Platinum-E cells produced nearly identical percentages of EGFP-positive cells in flow cytometry, indicating comparable infection rates for both plasmids (Fig. 5b, c; Supplementary Fig. 11). Similar results were observed when infecting the adherent murine NIH3T3 cell line (Supplementary Fig. 12). The reproducibility of these findings in two independent experiments, using retroviral supernatants from sequence-verified plasmid preparations, indicates that correcting the orientation of the *bla* ORF and part of the P3 promoter has no adverse effect on the retroviral properties of these plasmids.

## Discussion

Our findings elucidate unexpected challenges associated with the pBMN-I-GFP plasmid during bacterial propagation, providing a mechanistic explanation for impaired growth in *E. coli*. The identification of an inverted fragment containing the *bla* ORF and part of its promoter explains the observed failure of antibiotic resistance. Correcting the orientation of the AmpR ORF and P3 promoter fragment in pBMN-I-GFP and its derivatives restored both bacterial growth and *bla* activity, emphasizing the critical importance of proper gene orientation and integrity in plasmid design, particularly for antibiotic resistance genes used as selection markers. These results underscore the need for thorough sequence verification of commonly used plasmids - a process now greatly simplified by the widespread availability of affordable nanopore sequencing technologies, which offer read lengths of 10–100 kb or more^14^. This is particularly important given a recent large-scale survey of plasmids from hundreds of laboratories, which revealed that nearly half contained design and/or sequence errors^15^.

The growth impairment observed on carbenicillin or ampicillin agar plates was likely due to disrupted *bla* transcription caused by the inversion of the *Bsp*HI fragment, which contains the AmpR ORF and parts of the P3 promoter. This inversion interfered with *bla* expression, preventing the production of β-lactamase, the enzyme essential for inactivating many β-lactam antibiotics. We did not formally analyze transcription directionality around the *bla* gene or the presence of alternative or cryptic promoters in detail. However, we find it unlikely that these mechanisms play a significant role in the expression under our experimental conditions, as plasmids in the original orientation showed no β-lactamase activity above background levels in the nitrocefin assay. Restoring the correct orientation fully recovered both resistance and bacterial growth. Unintended changes at the *bla* promoter are not uncommon, as seen with e.g. plasmid pXP420, also distributed by Addgene, along with its corrected version, pXP420 v2^16^.

Importantly, the restored orientation of the *bla* ORF and P3 promoter fragment did not appear to negatively impact the retroviral functionality of the plasmids. Retroviral infection efficiencies of the murine pro-B cell line 38B9 and NIH3T3 cells remained comparable, indicating that the modification did not impair the retroviral packaging or transduction capabilities of the plasmids. This finding is particularly significant for researchers planning to utilize prBMN-I-EGFP and vectors derived from it for retroviral gene delivery, as it demonstrates that correcting antibiotic resistance issues does not compromise the plasmid’s utility in generating high-titer retroviral particles.

An unresolved question is why some *E. coli* cells carrying pBMN-I-GFP and its derivatives eventually grow on ampicillin- or carbenicillin-containing LB agar plates and medium after an extended incubation period. While not all colonies inoculated into selective liquid LB medium continue to grow or yield significant amounts of plasmid DNA after isolation, certain clones appear to replicate the plasmid successfully, enabling their propagation. ONT sequencing of a few of these clones did not reveal any alterations in the plasmids (data not shown). Another possibility is that compensatory mutations arise in these cells during prolonged incubation, allowing them to survive in selective media and maintain the plasmid at a relatively stable copy number.

Before identifying the underlying cause of the pBMN-I-GFP behavior, we occasionally achieved success by switching bacterial strains. We primarily used *E. coli* strains such as Stbl3, GeneHogs, and DH5α, with similar results across these strains. However, trying alternative strains like XL1-Blue or DH10B may be promising as well and should be evaluated on a case-by-case basis, particularly depending on the specific insert being cloned. When cloning into pBMN-I-GFP vectors, it is often necessary to generate multiple clones to screen for the correct one. Reducing the concentration of selective antibiotics, such as lowering ampicillin to 10 µg/mL or carbenicillin to 5 µg/mL, could also increase the number of clones, although this may result in clones with lower DNA yields during plasmid preparation.

In summary, this study emphasizes the critical importance of thoroughly characterizing laboratory materials, as even minor oversights can have significant impacts. By resolving the growth defects observed in *E. coli* cells carrying pBMN-I-GFP under selective conditions and successfully generating and characterizing a corrected version in prBMN-I-EGFP, we demonstrate retained retroviral function and ensure the continued utility of prBMN-I-EGFP as a valuable resource for future research.

## Materials and methods

### Cloning of retroviral vectors

#### Cloning of prBMN-I-EGFP

The ampicillin resistance cassette, the ampicillin promoter, the origin of replication and the 3’ LTR of the Moloney murine leukemia virus (MMLV) were amplified from the plasmid pBabe Puro^4^ using the primers Amp-ori SalI for (GATGTCGACGATAAAATAAAAGATTTTATTTAGTCTC) and Amp-ori SpeI rev (GATGACTAGTGCTGCAGGTGGCACTTTTCGGGGAA) and Invitrogen™ Accu-Prime™ Pfx DNA-Polymerase (Thermo Fisher Scientific, Dreieich, Germany). All primers mentioned in the Materials and methods section were ordered from Thermo Fisher Scientific and are shown in 5’ to 3’ orientation. The 2697 bp PCR product was excised from a 1.0% agarose gel, purified with the QIAGEN gel extraction kit (QIAGEN, Hilden, Germany) and eluted in buffer EB. The 5’ ends of the PCR product were then phosphorylated with T4 DNA Polynucleotide Kinase (NEB, Frankfurt, Germany), heat-inactivated for 20 min at 65 °C and self-ligated using T4 DNA Ligase (NEB). After electroporation of 1:20 volume of the ligation reaction into *E. coli* GeneHogs bacteria (Thermo Fisher Scientific, discontinued), cells were selected on LB/Amp agar plates at 37 °C overnight, single colonies were expanded, and isolated plasmid DNA was analyzed by restriction digests. Clones with the correct digestion pattern were sequenced (Macrogen Europe BV, Amsterdam, The Netherlands) using primers EGFP for (GAAGCGCGATCACATGGT), Amp scr rev (CGCTGTTGAGATCCAGTTCG), Amp sense (CGGGAAGCTAGAGTAAGTAGTTCG), PCR check Amp 3 for (GTATCATTGCAGCACTGGGG), pMB1-ori-screen-F (GTCTTACCGGGTTGGACTCA) and Amp middle seq for (CGGTCGCCGCATACACTATT). Correct clones were retransformed into heat shock-competent *E. coli* Stbl3 bacteria (Thermo Fisher Scientific) and named pAmpR – ori – 3’ LTR. This plasmid was digested with the restriction enzymes *Sal*I-HF and *Spe*I-HF (NEB), where “HF” stands for “high fidelity”. These engineered restriction endonucleases exhibit greater cleavage accuracy than their wild-type counterparts.A *Sal*I/*Spe*I fragment of plasmid pBMN-I-GFP (kind gift of Garry Nolan; also available as Addgene plasmid # 1736) containing the psi packaging signal, the IRES element as well as EGFP was ligated to the *Sal*I/*Spe*I-digested PCR product, yielding plasmid prBMN-I-EGFP delta 5’ LTR. Finally, this plasmid was linearized with *Spe*I-HF, dephosphorylated by treatment with Calf Intestinal Phosphatase (NEB) and ligated with the equally digested MMLV 5’ LTR fragment from pBMN-I-GFP. After electroporation of a 1:20 volume of the ligation reaction into *E. coli* GeneHogs bacteria, cells were selected on LB/Amp agar plates at 37 °C overnight, single colonies were grown in liquid LB/Amp medium, and isolated plasmid DNA was analyzed by restriction digests. Correct clones were retransformed into heat shock-competent *E. coli* Stbl3 bacteria. The complete plasmid sequence was confirmed using Oxford Nanopore Technologies (ONT) sequencing technology (Microsynth Seqlab GmbH, Göttingen, Germany) and designated prBMN-I-EGFP. For transfection into human Platinum-E cells, all plasmid DNAs were isolated using the QIAGEN Plasmid Maxi Kit (QIAGEN).

### Cloning of prBMN-I-EGFP-T2A-Puro

The plasmid pAmpR – ori – 3’ LTR was digested with restriction enzymes *Sal*I-HF and *Spe*I-HF and ligated with an equally digested fragment of plasmid pBMN-I-EGFP-T2A-Puro^11^ containing the psi packaging signal, the IRES element as well as an EGFP-T2A-Puro cassette. After electroporation of a 1:20 volume of the ligation reaction into *E. coli* GeneHogs bacteria, cells were selected on LB/Amp agar plates at 37 °C overnight, single colonies were grown in liquid LB/Amp medium, and isolated plasmid DNA was analyzed by restriction digests. Correct clones were designated prBMN-I-EGFP-T2A-Puro delta 5’ LTR. Finally, this plasmid was linearized with *Spe*I-HF, dephosphorylated by treatment with Calf Intestinal Phosphatase and ligated with the equally digested MMLV 5’ LTR fragment from pBMN-I-GFP. After electroporation of a 1:20 volume of the ligation reaction into *E. coli* GeneHogs bacteria, cells were selected on LB/Amp agar plates at 37 °C overnight, single colonies were grown in liquid LB/Amp medium, and isolated plasmid DNA was analyzed by restriction digests. Correct clones were retransformed into heat shock-competent *E. coli* Stbl3 bacteria. The complete plasmid sequence was confirmed using ONT sequencing technology and designated prBMN-I-EGFP-T2A-Puro. For transfection into human Platinum-E cells, all plasmid DNAs were isolated using the QIAGEN Plasmid Maxi Kit (QIAGEN).

### Heat-shock transformation of plasmid DNA in *E. coli*

The plasmids pBMN-I-GFP (Garry Nolan, unpublished), pBabe Puro^4^, pBMN-I-EGFP-T2A-Puro, pBMN-I-EGFP/Puro^11^, prBMN-I-EGFP-T2A-Puro, prBMN-I-GFP (this study), along with a plasmid-free control, were transformed into heat-shock competent *E. coli* bacteria prepared according to the method of^17^ with modifications. Briefly, a 5 mL seed culture of cells was grown to saturation in LB medium (Thermo Fisher Scientific) and diluted 1:100 in 50 mL fresh LB in a 500 mL conical flask. The diluted culture was incubated at 37 °C until an OD_600_ of 0.5 was reached. Eppendorf tubes (Eppendorf SE, Hamburg, Germany) and TSS buffer (for 50 mL, dilute 5 g PEG 8000 in 1.5 mL 1 M MgCl_2_ and add LB to 47.5 mL. Sterilize with a 0.22 μm filter and add 2.5 mL sterile DMSO) were chilled on ice. The culture was then divided into two 50 mL Falcon tubes and incubated on ice for 10 min. Cells were harvested by centrifugation at 3000 rpm for 10 min at 4 °C. The supernatant was carefully removed and cell pellets were resuspended in chilled TSS buffer (10% of the original culture volume). Aliquots of 100 µL were dispensed into pre-chilled Eppendorf tubes and stored at -80 °C until further use.

Twenty ng of plasmid DNA was transformed into 30 µL of heat-shock competent *E. coli* Stbl3 or DH5α (Thermo Fisher Scientific) cells. The cells were incubated on ice for 30 min, heat-shocked at 42 °C for 1 min, then placed on ice for 5 min before adding LB medium to a final volume of 1 mL. Cells were allowed to recover at 37 °C for 1 h with shaking at 1100 rpm on a ThermoMixer C (Eppendorf). The cells were briefly centrifuged for 10 s at 16,900× g, and all but 25 µL was carefully removed with a pipette tip. The small bacterial pellet was then resuspended and streaked with a pipette tip on sectors on 100-mm LB agar or LB agar + carbenicillin (Carl Roth GmbH + Co. KG, Karlsruhe, Germany, 50 µg/mL) or LB agar + ampicillin (Carl Roth GmbH + Co. KG, 100 µg/mL) plates (Greiner Bio-One, Frickenhausen, Germany). Plates were incubated overnight at 37 °C for 20 h and imaged using a BioStep Argus X1 gel documentation system (BioStep, Burkhardtsdorf, Germany). The complete plasmid sequences of all plasmids used in this study were confirmed using ONT sequencing.

### Visualization of β-lactamase activity

Heat-shock competent *E. coli* Stbl3 cells were transformed as described above with either no plasmid DNA (mock) or one of the following retroviral plasmids: pBabe Puro, pBMN-I-EGFP/Puro, pBMN-I-EGFP-2A-Puro, prBMN-I-EGFP-T2A-Puro, pBMN-I-GFP, or prBMN-I-EGFP. The transformed cells were inoculated into LB medium containing 50 µg/mL carbenicillin and grown at 37 °C until reaching stationary phase. BD BBL Cefinase discs (Fisher Scientific GmbH, Schwerte, Germany) were placed in an empty Petri dish and moistened with a drop of purified water. A 10 µL aliquot of bacterial suspension was applied to the disc surface, and color change was observed. A red color indicated β-lactamase activity, while a yellow color indicated no β-lactamase activity. Images were captured 5 min after the application.

### Cell lines and culture conditions

Murine 38B9 pro-B cells^12^ were cultured in RPMI 1640 medium supplemented with 10% fetal bovine serum, 1% L-glutamine, 1% sodium pyruvate, 1% penicillin/streptomycin and 0.1% β-mercaptoethanol (all from Thermo Fisher Scientific) at 37 °C and 5% CO_2_ in a humidified incubator. The murine fibroblast cell line NIH3T3 (ATCC cat. no. CRL-1658;^18^) and the ecotropic human packaging cell line Platinum-E (Cell Biolabs, Inc., San Diego, CA, USA;^13^) were grown in DMEM medium supplemented with 10% fetal bovine serum, 1% L-glutamine and 1% penicillin/streptomycin (all from Thermo Fisher Scientific) and maintained at 37 °C and 7.5% CO_2_ in a humidified incubator.

### Transient transfection of Platinum-E cells for retrovirus production

24 hours before transfection, 7.8 × 10^6^ Platinum-E cells were plated in 20 mL complete DMEM medium in 150-mm dishes (Greiner Bio-One). For transient transfection on the next day, an Eppendorf cup was prepared with 27.5 µg of retroviral expression vector diluted in a total volume of 479 µL OptiMEM medium without supplements (Thermo Fisher Scientific) and 110 µL PEI (Polyethylenimine ”Max”, (Mw 40,000) - High Potency Linear PEI, 1 mg/mL; Polysciences Inc., Warrington, PA, USA) was added. After brief vortexing, the mixture was incubated at room temperature for 10 min and added dropwise to Platinum-E cells. After 5 h of incubation at 37 °C, the DNA/PEI/DMEM medium was carefully removed and replaced with 15 mL complete DMEM medium. The supernatant was collected 72 h after transfection, filtered through 0.45-µm syringe filters (Sartorius, Göttingen, Germany) and either used directly for retroviral infection or stored at -80 °C until use. To determine the transfection efficiency, Platinum-E cells were carefully washed with PBS after removal of the retroviral supernatant, trypsinized and analyzed for EGFP fluorescence by flow cytometry (Supplementary Fig. 10).

### Retroviral infection of 38B9- and NIH3T3 cells

Retroviral infection was performed by spinoculation^11^. Briefly, 38B9 suspension cells were counted and 1 × 10^6^ cells harvested by centrifugation. Cell pellets were resuspended in 1 mL retroviral culture supernatant containing 4 µg/mL polybrene (Sigma-Aldrich, Taufkirchen, Germany) and transferred to a 24-well plate. For NIH3T3 cells, 2.5 × 10^4^ cells were seeded in 1 mL complete DMEM medium in a 24-well plate (Greiner Bio-One) one day before infection. On the day of infection, the medium was removed and replaced with 1 mL retroviral culture supernatant from Platinum-E cells transfected with the respective vectors plus 4 µg/mL polybrene. Both cell lines were centrifuged at 1700× g for 3.5 h at 33 °C to achieve close contact between the retroviral particles and the cells. Retroviral supernatants were then removed and cell culture was continued in fresh medium under the appropriate conditions. Live cells were analyzed for EGFP expression by flow cytometry on the day of infection and every day thereafter for up to 3 days.

### Flow cytometry

Trypsinized transfected Platinum-E, trypsinized and infected NIH3T3 cells, and infected 38B9 suspension cells were analyzed using a Gallios flow cytometer (Beckman Coulter, Krefeld, Germany). Scattered light was measured by the forward- (FS INT) and sideward (SS INT) scatter detectors to identify presumably viable cells. A plot of the FS peak signal (FS PEAK) against the area based FS intensity (FS INT) allowed the exclusion of multimeric cell agglomerates. EGFP fluorescence intensity was measured in the FL-1 channel. Raw data were analyzed using Kaluza Analysis software version 2.3 (Beckman Coulter).

## Electronic supplementary material

Below is the link to the electronic supplementary material.

Supplementary Material 1

## Data Availability

Data supporting the results described in this manuscript may be obtained from the corresponding author upon reasonable request.
