# Supplementary material for: Correcting promoter and beta-lactamase ORF orientation in a widely-used retroviral plasmid to restore bacterial growth
Source: Sci Rep. 2025 Mar 11;15:8348. doi: 10.1038/s41598-025-93222-y (PMC11897316; doi:10.1038/s41598-025-93222-y)
Supplement: Supplementary file 1 — Supplementary Material 1 [file 41598_2025_93222_MOESM1_ESM.docx]

# Supplementary Information

Correcting Promoter and Beta-Lactamase ORF Orientation in a Widely-Used Retroviral Plasmid to Restore Bacterial Growth

Jürgen Wittmann^1*^

^1^Division of Molecular Immunology, Department of Internal Medicine III, Nikolaus-Fiebiger-Center of Molecular Medicine (NFZ), Friedrich-Alexander-Universität Erlangen-Nürnberg (FAU), Erlangen, Germany

*** Correspondence:**Corresponding Author
juergen.wittmann@uk-erlangen.de

**This PDF file includes:**

Supplementary Fig. 1. Plasmid map of pBMN-I-GFP (according to information on website).

Supplementary Fig. 2. Growth of *E. coli* after transformation with retroviral plasmids pBMN-I-GFP and pBabePuro on LB/Ampicillin and LB agar plates.

Supplementary Fig. 3. Notice on Addgene web site for pBMN-I-GFP (Plasmid #1736).

Supplementary Fig. 4. Alignment of available sequence data for the retroviral plasmid pBMN-I-GFP.

Supplementary Fig. 5. Plasmid map of pBMN-I-GFP (accoding to ONT sequencing).

Supplementary Fig. 6. Plasmid map of pBabe Puro.

Supplementary Fig. 7. Proposed mechanism of how the *bla* gene affects bacterial growth before and after orientation reversal.

Supplementary Fig. 8. Plasmid map of prBMN-I-EGFP.

Supplementary Fig. 9. Plasmid map of prBMN-I-EGFP-T2A-Puro.

Supplementary Fig. 10. Representative flow cytometric gating strategy for EGFP fluorescence of Platinum-E cells transfected with retroviral constructs pBMN-I-GFP and prBMN-I-EGFP.

Supplementary Fig. 11. Representative flow cytometric gating strategy for EGFP fluorescence in 38B9 cells infected with retroviral supernatants derived from pBMN-I-GFP and prBMN-I-EGFP.

Supplementary Fig. 12. Flow cytometric analyses for EGFP fluorescence in NIH3T3 cells infected with retroviral supernatants derived from pBMN-I-GFP and prBMN-I-EGFP.


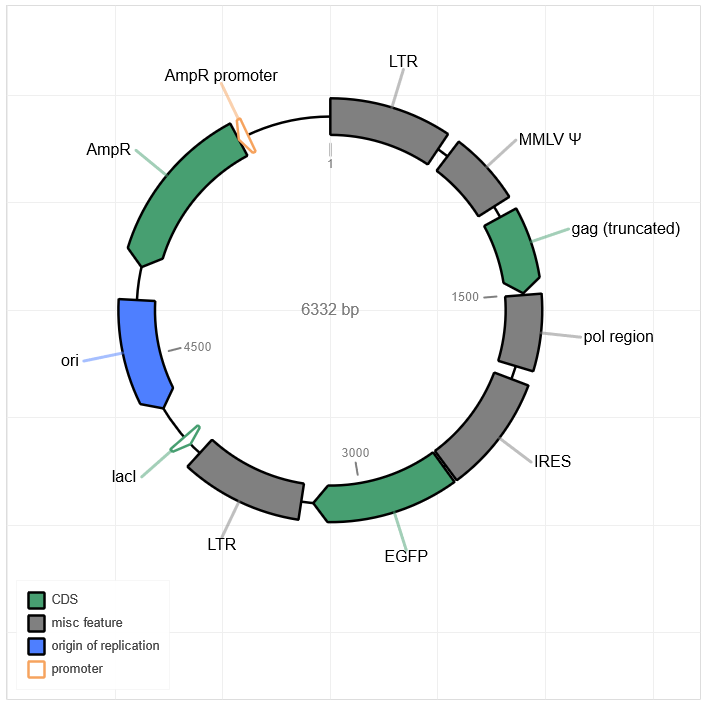


**Supplementary Figure 1. Plasmid map of pBMN-I-GFP (according to information on website).**

Vector map for plasmid pBMN-I-GFP (6332 bp, https://web.stanford.edu/group/nolan/_OldWebsite/plasmid_maps/pBMN-i-GFP%20seq.doc, accessed on October 23, 2024). Plasmid features: Moloney Murine Leukemia Virus (MMLV) 5‘ long terminal repeat (LTR, bp 1 – 594), packaging signal of MMLV (MMLV Ψ, bp 656 – 1014), truncated MMLV gag gene lacking the start codon [gag (truncated), bp 1078 – 1495], MMLV pol region containing the splice acceptor site (pol region, bp 1504 – 1878), encephalomyocarditis virus (EMCV) internal ribosome entry site (IRES, bp 1949 – 2522), enhanced green fluorescent protein (EGFP, bp 2535 – 3255), MMLV 3‘ long terminal repeat (LTR, bp 3318 – 3912), lac repressor [lacI, bp 3979 – 4028 (C)], pBR322 origin of replication [ori, bp 4213 – 4802 (C)], Ampicillin-resistance gene beta-lactamase (AmpR, bp 4975 – 5836), Ampicillin-resistance gene promoter [AmpR promoter, bp 5837 – 5889 (C)]. (C): element on complementary strand, CDS: coding sequence. The plasmid map was created with pLannotate [19].


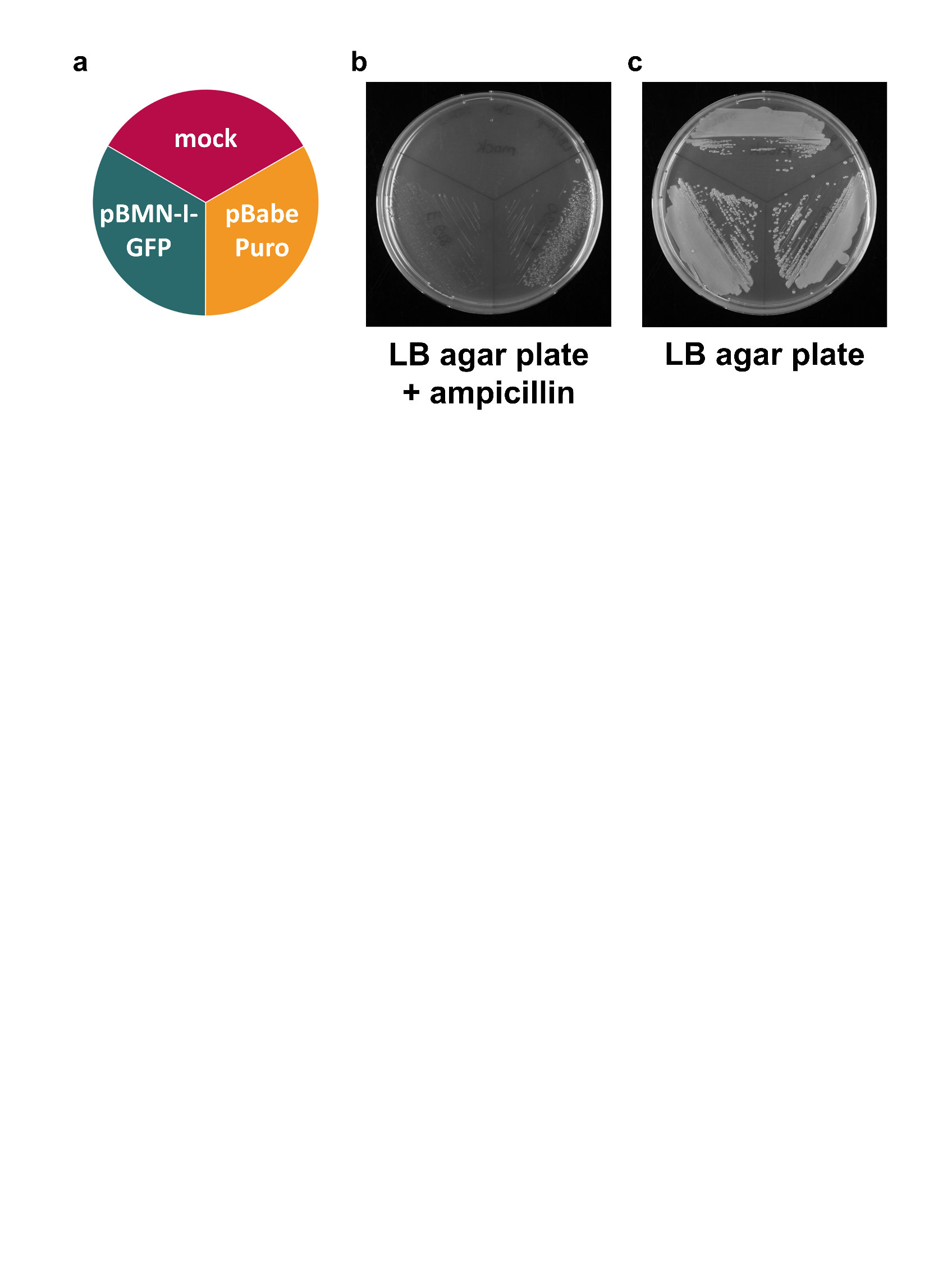


**Supplementary Figure 2. Growth of *E. coli* after transformation with retroviral plasmids pBMN-I-GFP and pBabePuro on LB/Ampicillin and LB agar plates.**

Heat-shock competent *E.* *coli* Stbl3 cells were transformed with either no plasmid DNA (mock) or with plasmids pBMN-I-GFP or pBabe Puro and streaked as shown in (**a**) onto (**b**) an LB agar plate containing 100 µg/mL ampicillin or (**c**) an LB agar plate without antibiotics. Plates were incubated at 37°C for 20 hours, and images were captured. The image of the LB agar plate without antibiotics is identical to that shown in Figure 1c Results are representative of three independent experiments.


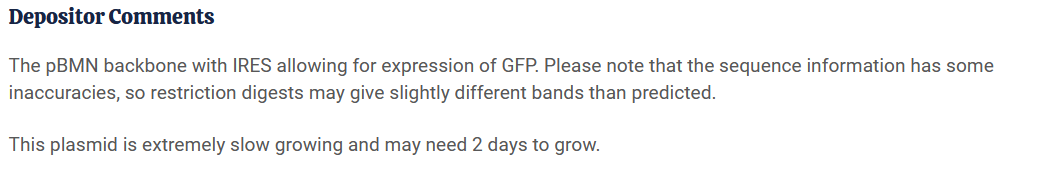


**Supplementary Figure 3. Notice on Addgene web site for pBMN-I-GFP (Plasmid #1736).**

Screenshot of a comment of the depositor on the Addgene website under https://www.addgene.org/1736/ (accessed on October 23, 2024).


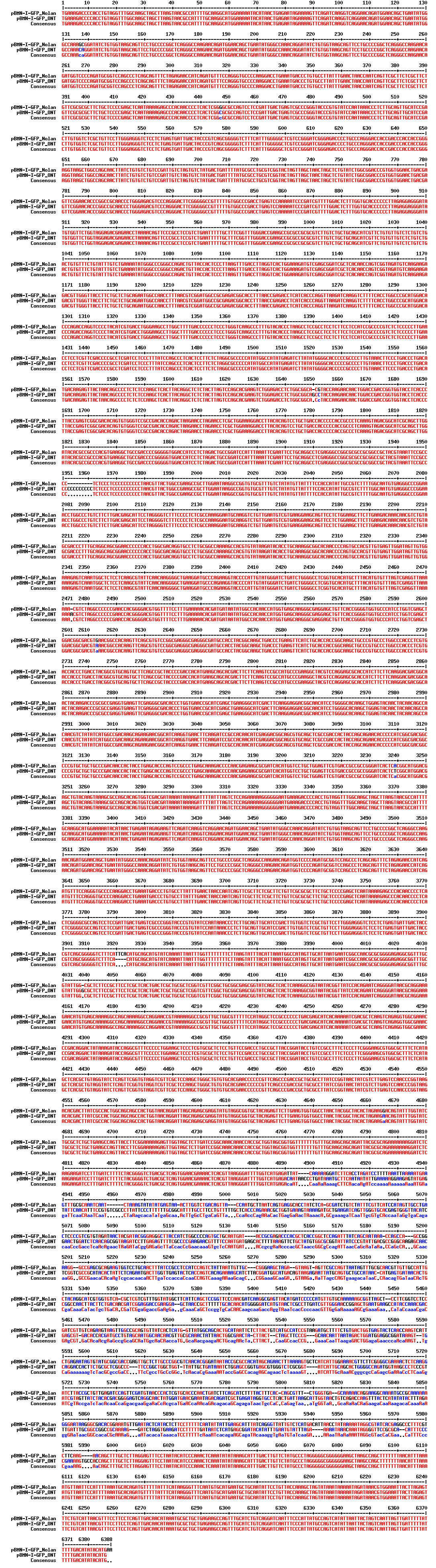


**Supplementary Figure 4. Alignment of available sequence data for the retroviral plasmid pBMN-I-GFP.**

The plasmid sequence of pBMN-I-GFP from the Nolan web site (https://web.stanford.edu/group/nolan/_OldWebsite/retroviral_systems/phx.html; accessed on October 23, 2024; "pBMN-I-GFP_Nolan") was aligned with sequences obtained via Oxford Nanopore Technology (ONT) sequencing (“pBMN-I-GFP_ONT”). Plasmid DNA sequences were aligned using MultAlin [6; http://multalin.toulouse.inra.fr/multalin/, accessed on October 23, 2024].


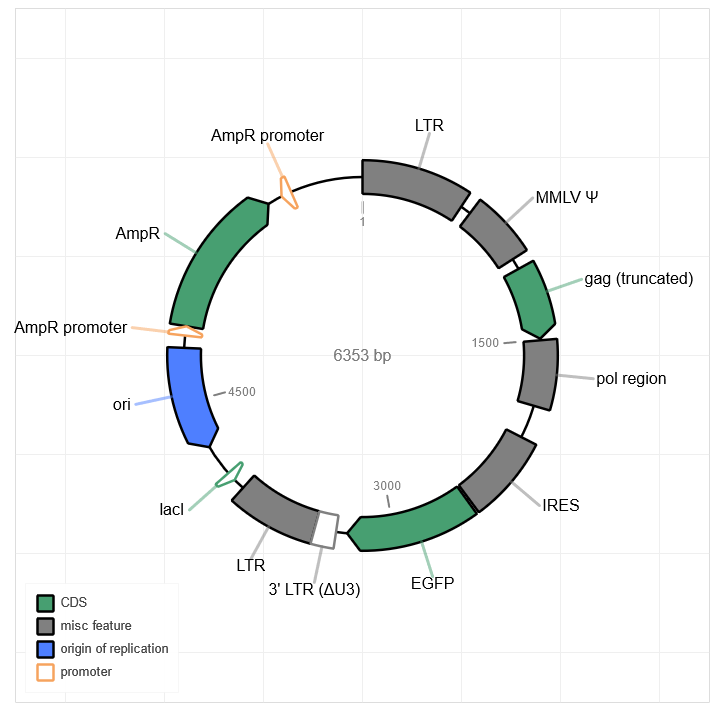


**Supplementary Figure 5. Plasmid map of pBMN-I-GFP (accoding to ONT sequencing).**

Vector map for plasmid pBMN-I-GFP (6353 bp). Plasmid features: Moloney Murine Leukemia Virus (MMLV) 5‘ long terminal repeat (LTR, bp 1 – 592), packaging signal of MMLV (MMLV Ψ, bp 654 – 1012), truncated MMLV gag gene lacking the start codon [gag (truncated), bp 1076 – 1493], MMLV pol region containing the splice acceptor site (pol region, bp 1502 – 1877), encephalomyocarditis virus (EMCV) internal ribosome entry site (IRES, bp 2068 – 2531), enhanced green fluorescent protein (EGFP, bp 2544 – 3264), self-inactivating 3' long terminal repeat from MMLV [3‘ LTR (ΔU3), bp 3327 – 3449], MMLV 3‘ long terminal repeat (LTR, bp 3449 – 3919), lac repressor [lacI, bp 3984 – 4037 (C)], pBR322 origin of replication [ori, bp 4219 – 4808 (C)], Part of Ampicillin-resistance gene promoter (AmpR promoter, bp 4878 – 4931), Ampicillin-resistance gene beta-lactamase (AmpR, bp 4931 – 5792), Part of Ampicillin-resistance gene promoter [AmpR promoter, bp 5877 – 5937 (C)]. (C): element on complementary strand, CDS: coding sequence. The plasmid map was created with pLannotate [19].


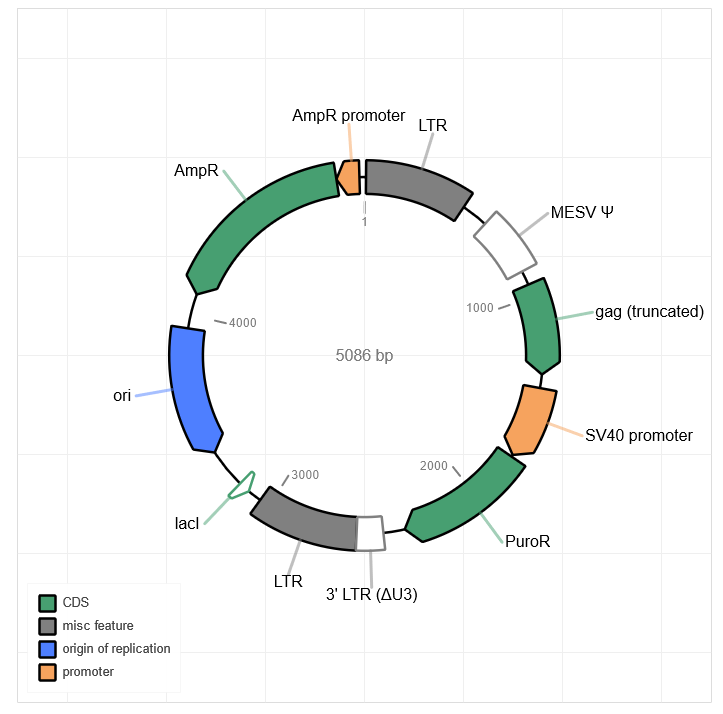


**Supplementary Figure 6. Plasmid map of pBabe Puro.**

Vector map for plasmid pBabe Puro (5086 bp, **[Morgenstern and Lund, 1990]**). Plasmid features: Moloney Murine Leukemia Virus (MMLV) 5‘ long terminal repeat (LTR, bp 7 – 477), packaging signal of murine embryonic stem cell virus (MESV Ψ, bp 600 – 875), truncated MMLV gag gene lacking the start codon [gag (truncated), bp 941 – 1358], SV40 enhancer and early promoter (SV40 promoter, bp 1420 – 1750), puromycin N-acetyltransferase (PuroR, bp 1759 – 2359), self-inactivating 3' long terminal repeat from MMLV [3‘ LTR (ΔU3), bp 2457 – 2579], MMLV 3‘ long terminal repeat (LTR, bp 2579 – 3049), lac repressor [lacI, bp 3114 – 3167 (C)], pBR322 origin of replication [ori, bp 3349 – 3938 (C)], Ampicillin-resistance gene beta-lactamase [AmpR, bp 4097 – 4958 (C)], Ampicillin-resistance gene promoter [AmpR promoter, bp 4958 – 5063 (C)]. (C): element on complementary strand, CDS: coding sequence. The plasmid map was created with pLannotate [19].


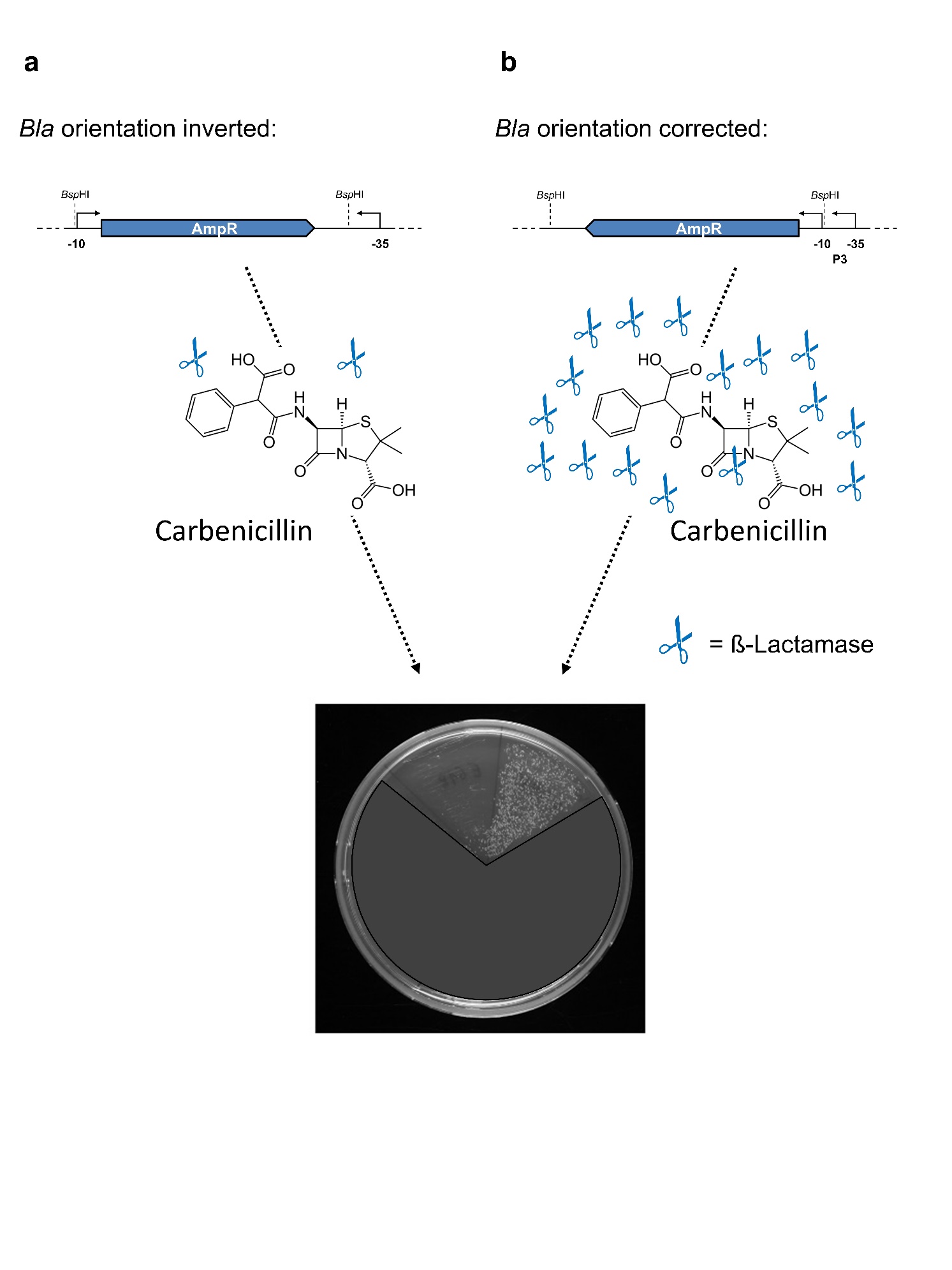


**Supplementary Figure 7. Proposed mechanism of how the *bla* gene affects bacterial growth before and after orientation reversal.**

(**a**) Inversion of *bla* and parts of its promoter may reduce *bla* transcription, leading to lower β-lactamase synthesis. As a result, the β-lactam ring of antibiotics such as ampicillin or carbenicillin is not efficiently cleaved, preventing bacterial growth on antibiotic-containing media. (**b**) When the correct orientation is restored, *bla* transcription and translation resume, increasing β-lactamase production. This enhances antibiotic inactivation, allowing bacteria carrying this plasmid to grow on LB agar plates containing carbenicillin.


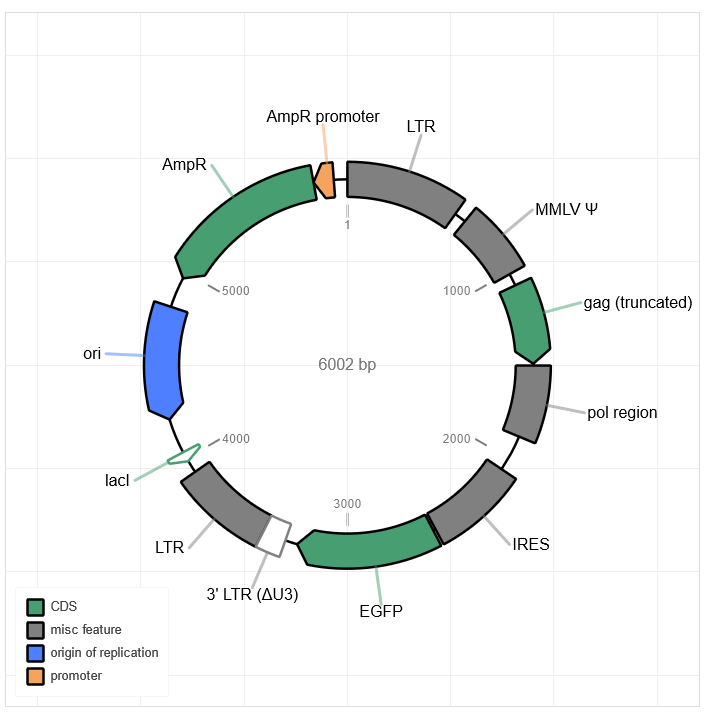


**Supplementary Figure 8. Plasmid map of prBMN-I-EGFP.**

Vector map for plasmid prBMN-I-EGFP (6002 bp, this work). Plasmid features: Moloney Murine Leukemia Virus (MMLV) 5‘ long terminal repeat (LTR, bp 1 – 592), packaging signal of MMLV (MMLV Ψ, bp 654 – 1012), truncated MMLV gag gene lacking the start codon [gag (truncated), bp 1076 – 1493], MMLV pol region containing the splice acceptor site (pol region, bp 1502 – 1877), encephalomyocarditis virus (EMCV) internal ribosome entry site (IRES, bp 2067 – 2530), enhanced green fluorescent protein (EGFP, bp 2544 – 3263), self-inactivating 3' long terminal repeat from MMLV [3‘ LTR (ΔU3), bp 3326 – 3448], MMLV 3‘ long terminal repeat (LTR, bp 3448 – 3918), lac repressor [lacI, bp 3983 – 4036 (C)], pBR322 origin of replication [ori, bp 4218 – 4807 (C)], Ampicillin-resistance gene beta-lactamase [AmpR, bp 4966 – 5827 (C)], Ampicillin-resistance gene promoter [AmpR promoter, bp 5827 – 5932 (C)]. (C): element on complementary strand, CDS: coding sequence. The plasmid map was created with pLannotate [19].


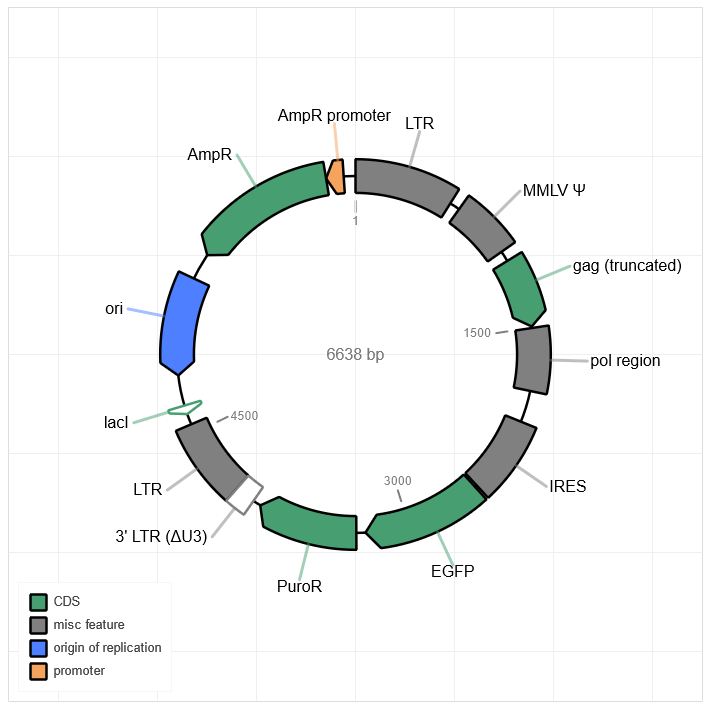


**Supplementary Figure 9. Plasmid map of prBMN-I-EGFP-T2A-Puro.**

Vector map for plasmid prBMN-I-EGFP-T2A-Puro (6638 bp, this work). Plasmid features: Moloney Murine Leukemia Virus (MMLV) 5‘ long terminal repeat (LTR, bp 1 – 592), packaging signal of MMLV (MMLV Ψ, bp 654 – 1012), truncated MMLV gag gene lacking the start codon [gag (truncated), bp 1076 – 1493], MMLV pol region containing the splice acceptor site (pol region, bp 1502 – 1877), encephalomyocarditis virus (EMCV) internal ribosome entry site (IRES, bp 2067 – 2530), enhanced green fluorescent protein (EGFP, bp 2543 – 3260), T2A peptide (not shown in map, bp 3261 – 3314), puromycin N-acetyltransferase (PuroR bp 3315 – 3914), self-inactivating 3' long terminal repeat from MMLV [3‘ LTR (ΔU3), bp 3962 – 4084], MMLV 3‘ long terminal repeat (LTR, bp 4084 – 4554), lac repressor [lacI, bp 4619 – 4672 (C)], pBR322 origin of replication [ori, bp 4854 – 5443 (C)], Ampicillin-resistance gene beta-lactamase [AmpR, bp 5602 – 6463 (C)], Ampicillin-resistance gene promoter [AmpR promoter, bp 6463 – 6568 (C)]. (C): element on complementary strand, CDS: coding sequence. The plasmid map was created with pLannotate [19].


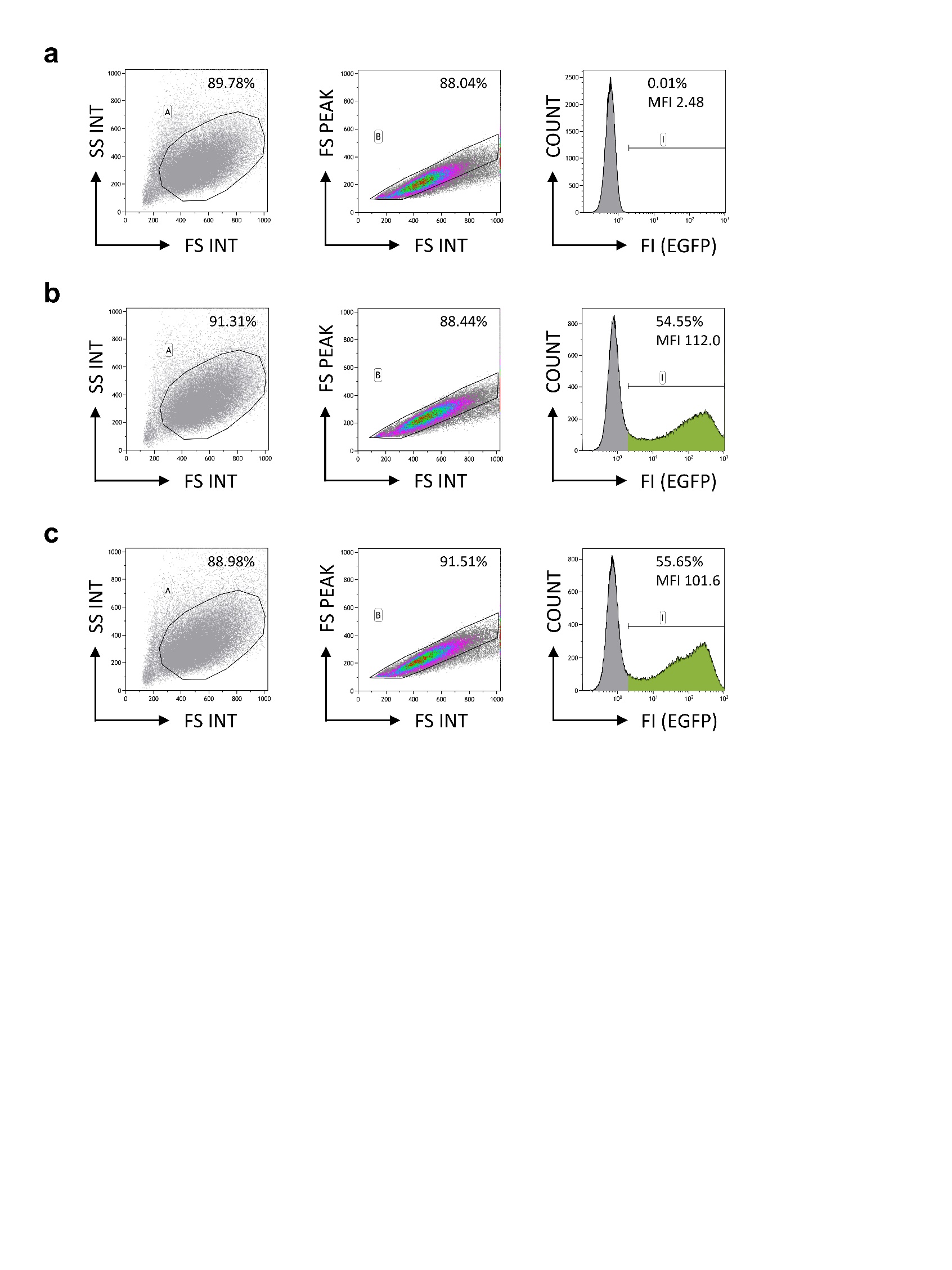


**Supplementary Figure 10. Representative flow cytometric gating strategy for EGFP fluorescence of Platinum-E cells transfected with retroviral constructs pBMN-I-GFP and prBMN-I-EGFP.**

Platinum-E cells were (**a**) mock transfected or transfected with plasmid DNA derived from (**b**) pBMN-I-GFP or (**c**) prBMN-I-EGFP, and incubated for three days before flow cytometric analysis to determine transfection efficiencies. For the hierarchical sequences of analysis (i.e. gating strategy), the scattered light intensity was measured by the forward scatter- (FS INT) and sideward scatter (SS INT) detectors, and presumably viable cells are displayed in gate “A” (1^st^ column). A plot of the FS peak signal (FS PEAK) against the area based FS intensity (FS INT) allowed to exclude multimeric cell agglomerates, and single cells are displayed in gate “B” (2^nd^ column). Cells present in gates “A” and “B” (COUNT) are then displayed in histogram plots for EGFP [FI (EGFP), gate “I”, 3^rd^ column] fluorescence intensities. Percentages of gated cells and mean fluorescence intensities (MFIs) of EGFP-positive cells are indicated in the graphs. In gates “A” and “B”, only 25% of events are shown. Data were collected with a Gallios flow cytometer and analyzed with Kaluza software. Results are representative of two independent experiments, each performed with independently isolated plasmids.


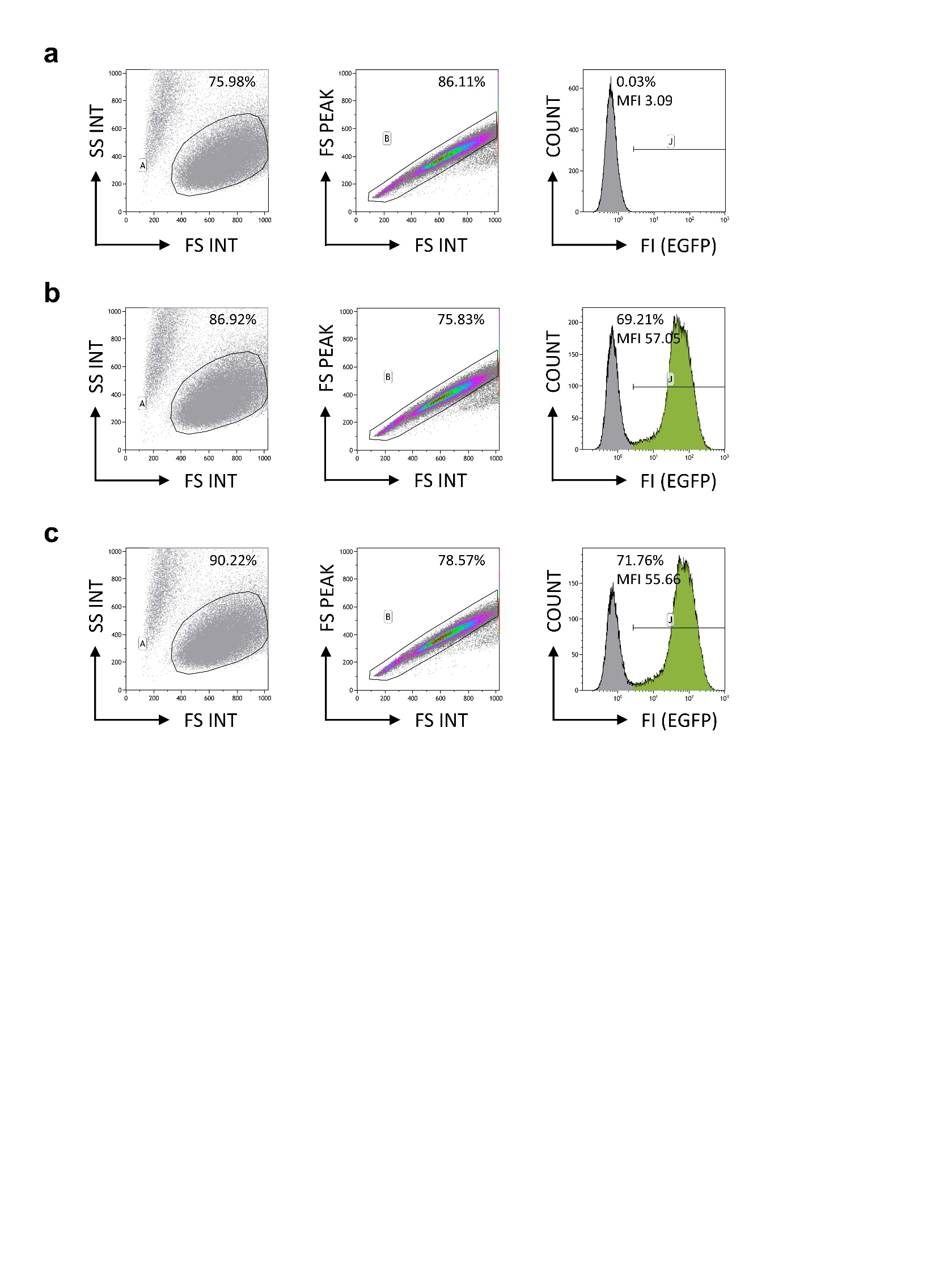


**Supplementary Figure 11. Representative flow cytometric gating strategy for EGFP fluorescence in 38B9 cells infected with retroviral supernatants derived from pBMN-I-GFP and prBMN-I-EGFP.**

Exemplary raw data for some analyses shown in Figure 5. 38B9 cells were (**a**) mock infected or infected with retroviral supernatants derived from (**b**) pBMN-I-GFP or (**c**) prBMN-I-EGFP, and incubated for two days before flow cytometric analysis to determine infection efficiencies. For the hierarchical sequences of analysis (i.e. gating strategy), the scattered light intensity was measured by the forward scatter- (FS INT) and sideward scatter (SS INT) detectors, and presumably viable cells are displayed in gate “A” (1^st^ column). A plot of the FS peak signal (FS PEAK) against the area based FS intensity (FS INT) allowed to exclude multimeric cell agglomerates, and single cells are displayed in gate “B” (2^nd^ column). Cells present in gates “A” and “B” (COUNT) are then displayed in histogram plots for EGFP- [FI (EGFP), gate “J”, 3^rd^ column] fluorescence intensities. Percentages of gated cells and mean fluorescence intensities (MFIs) of EGFP-positive cells are indicated in the graphs. Data were collected with a Gallios flow cytometer and analyzed with Kaluza software. Results are representative of two independent experiments, each performed using retroviral supernatant generated from independently isolated plasmids.


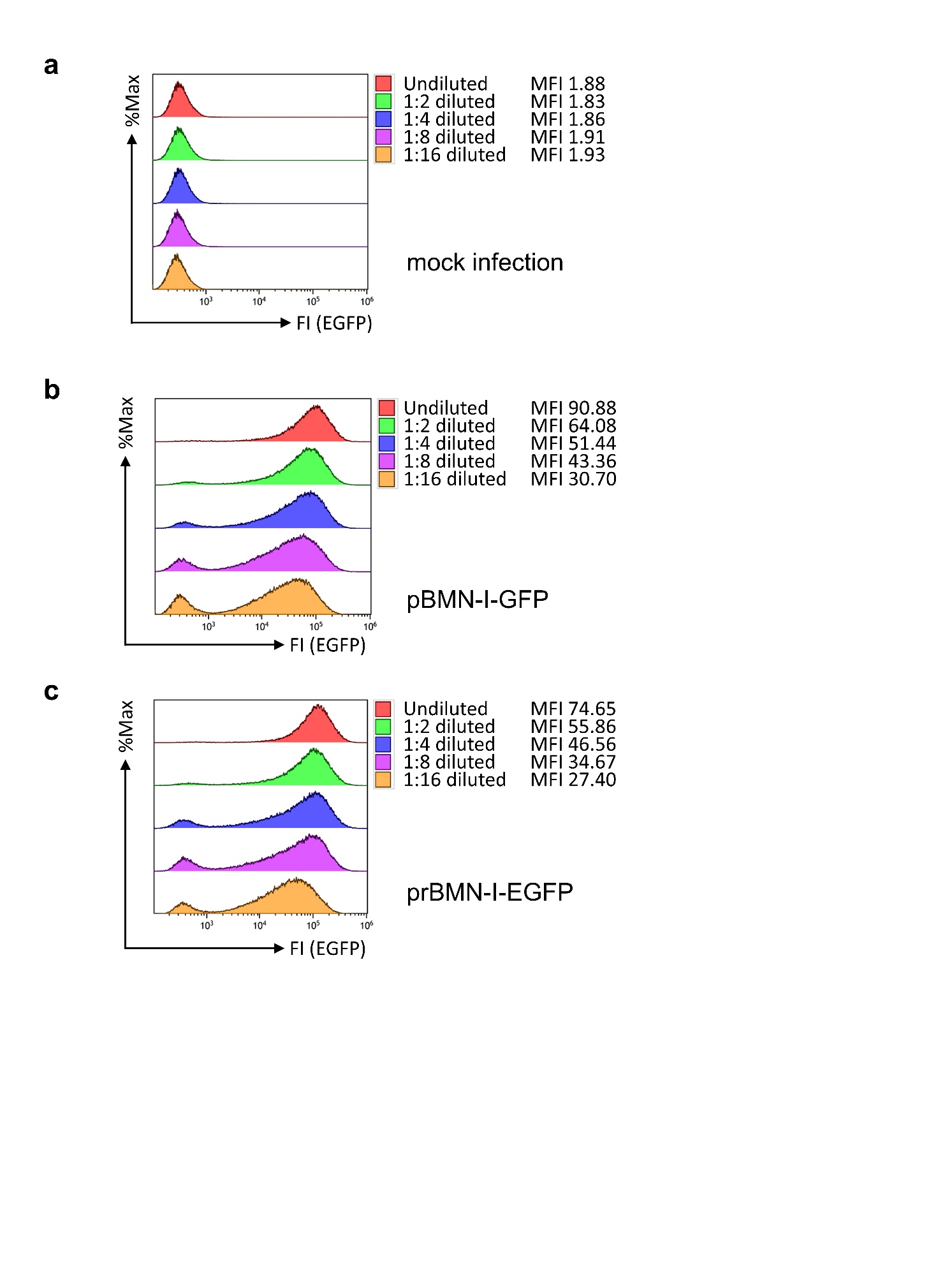


**Supplementary Figure 12. Flow cytometric analyses for EGFP fluorescence in NIH3T3 cells infected with retroviral supernatants derived from pBMN-I-GFP and prBMN-I-EGFP.**

NIH3T3 cells were infected with retroviral supernatants from (**a**) mock-, (**b**) pBMN-I-GFP- or (**c**) prBMN-I-EGFP-transfected Platinum-E cells at various dilution steps. EGFP fluorescence intensities, used to assess infection efficiency, were measured by flow cytometry two days after infection. Data acquisition and gating strategy followed those described in Supplemental figure 10. EGFP fluorescence intensities of live, single cells are presented as overlay histograms, with relative cell numbers normalized and presented as %Max. The mean fluorescence intensities (MFIs) of the EGFP-positive populations are indicated next to the labels. The results are representative of two independent experiments, each performed using retroviral supernatant generated from independently isolated plasmids.

# Supplementary Reference

1. McGuffie, M. J. & Barrick, J. E. pLannotate: engineered plasmid annotation. *Nucleic Acids Research* **49**, W516–W522 (2021).
